# Gender-Related Differences in Cerebrovascular Reactivity to L–Arginine in Middle-Aged Type 1 Diabetes Patients

**DOI:** 10.3390/ijms27041662

**Published:** 2026-02-09

**Authors:** Grzegorz M. Kozera, Jolanta Neubauer-Geryk, Bogumił Wolnik, Sebastian Szczyrba, Leszek Bieniaszewski

**Affiliations:** 1 Medical Simulation Centre, Medical University of Gdansk, 80-210 Gdansk, Poland; jolaneub@gumed.edu.pl (J.N.-G.); leszek.bieniaszewski@gumed.edu.pl (L.B.); 2 Department of Hypertension and Diabetology, Medical University of Gdansk, 80-210 Gdansk, Poland; 3 Department of Neurology, Medical University of Gdansk, 80-210 Gdansk, Poland

**Keywords:** cerebral microangiopathy, gender, vasomotor reactivity, middle cerebral artery, type 1 diabetes, transcranial Doppler, L–arginine

## Abstract

Type 1 diabetes (T1D) increases the risk for cerebral microangiopathy. However, the association between gender and cerebral microcirculatory dysfunction in T1D remains undetermined. Therefore, we have conducted a comparative analysis of cerebral endothelial-mediated microcirculatory parameters between middle-aged Caucasian females and males with type 1 diabetes. The present study examined the nitric oxide-induced vasomotor reactivity of middle cerebral arteries (MCA using transcranial Doppler and L–arginine infusion (L–arg VMR)). The study compared L–arg VMR between 23 males and 26 females with type 1 diabetes without a history of overt cerebrovascular disease. Mean L–arg VMR and baseline MCA flow velocity (V rest) were higher in females than in males (20.1 ± 5.4 vs. 14.6 ± 7.1% *p* < 0.01 and 73, 54–106 vs. 60.7–77 cm/s *p* < 0.01, respectively). Males were older than females (39.7 [range: 31.3–55.7] vs. 36.5 [range: 25.0–45.5] years, *p* = 0.02) and were characterized by later T1D onset and higher insulin/24 h, triglyceride levels and body mass index (BMI). Higher L–arg VMR in females persisted when co-variated with patients’ age, age of onset, BMI, triglyceride level and V rest. Cerebral vasomotor reactivity to L–arginine showed greater efficacy in middle-aged females than in males with T1D, independent of age and disease course. The protective effect of the female gender on cerebral endothelium function has been demonstrated in type 1 diabetes.

## 1. Introduction

Individuals diagnosed with type 1 diabetes (T1D) are at high risk for the early onset of both cerebral and systemic microvascular injuries. The presence of cerebral microcirculatory dysfunction in both premature and adult T1D patients has been demonstrated by impaired cerebral autoregulation or increased pulsatility indices in cerebral arteries, as shown in transcranial Doppler (TCD) examination [1,2,3,4,5]. Recent studies employing near-infrared spectroscopy have revealed subtle disturbances in cortical microcirculation. These alterations were reflected in changes to regional brain hemodynamic responses following increased physical exercise and visual stimulation [6,7]. Moreover, a recent report revealed rapid progression of structural microangiopathic changes on magnetic resonance imaging in middle-aged T1D subjects [8].

The severity of dysfunction in cerebral microcirculation in individuals with T1D is influenced by several factors, including the patient’s age, diabetes duration, and the presence of complications such as diabetic ketoacidosis and systemic microangiopathy, which includes overt nephropathy and retinal injury [1,2,4,9]. Small vessel disease plays a significant role in the etiology of stroke among type 1 diabetes patients [10,11]. Studies examining gender-related differences in cerebrovascular reactivity within this population have been limited. A noteworthy study conducted in 2001 reported no significant differences in acetazolamide-induced cerebrovascular reactivity between male and female adults with T1D [12]. Reports on systemic microangiopathy and gender in type 1 diabetes have not adequately examined cerebral microvasculature, leading to inconsistent results [13,14,15,16,17,18]. It would be advantageous to clarify the impact of gender on the extent of cerebral microangiopathy, as this could aid in developing early stroke prevention strategies for individuals with type 1 diabetes. Numerous studies highlight distinct cerebrovascular risks between middle-aged Caucasian men and women in the general population [19,20,21,22].

Transcranial Doppler is a noninvasive ultrasound technique utilized to evaluate cerebral blood flow function. This method enables early detection of cerebral microcirculatory disorders by evaluating cerebrovascular reactivity (CVR) [23,24,25,26,27,28]. Research has demonstrated that CVR is diminished in asymptomatic patients with cerebrovascular risk factors, individuals with psoriasis or silent white matter lesions, and those with overt lacunar infarctions or symptomatic carotid artery occlusion [29,30,31,32,33,34]. In most studies, the assessment of CVR has primarily been conducted using techniques that stimulate vascular function by altering serum partial carbon dioxide (CO_2_) pressure, typically through acetazolamide infusion or hyperventilation/breath-holding tests. Additionally, it is feasible to assess endothelium-dependent cerebral vasomotor reactivity (VMR) by increasing nitric oxide (NO) secretion through intravenous L–arginine infusion. A reduction in vasomotor reactivity to L–arginine (L–arg VMR) is considered a significant indicator of cerebral endothelial dysfunction [35,36]. This measure has also been utilized to assess the impact of various pharmacological interventions in individuals with vascular risk factors [37,38,39]. There is currently a notable deficiency in the literature concerning studies that specifically investigate VMR through the L–arginine infusion test in patients diagnosed with type 1 diabetes.

In this study, we retrospectively compared vasomotor reactivity to L–arginine evaluated by Transcranial Doppler between middle-aged females and males diagnosed with type 1 diabetes mellitus who do not exhibit overt cerebrovascular disease.

## 2. Results

### 2.1. A Comparison of the Median L–Arg VMR and Baseline MCA Flow Velocity

The medians of vasomotor reactivity to L–arginine and baseline middle cerebral artery (MCA) flow velocity were higher in females than in males with type 1 diabetes, as shown in Table 1. Women were younger and had an earlier onset of type 1 diabetes (T1D). They also exhibited lower insulin levels over 24 h, lower triglyceride (TG) serum levels, a lower body mass index (BMI), and experienced overweight or obesity less frequently than men.

The analysis revealed no significant differences between males and females regarding diabetes duration, HbA_1C_ levels, mean blood pressure, the presence of hyperlipidemia and arterial hypertension, cigarette smoking, or the incidence of nephropathy, retinopathy, and neuropathy. Furthermore, there were no differences in the use of statins or ACE inhibitors/ARBs between the two groups (Table 1).

### 2.2. Correlations Between L–Arg VMR and Patients’ Biometrics and Biochemical Parameters

**Figure 1:**
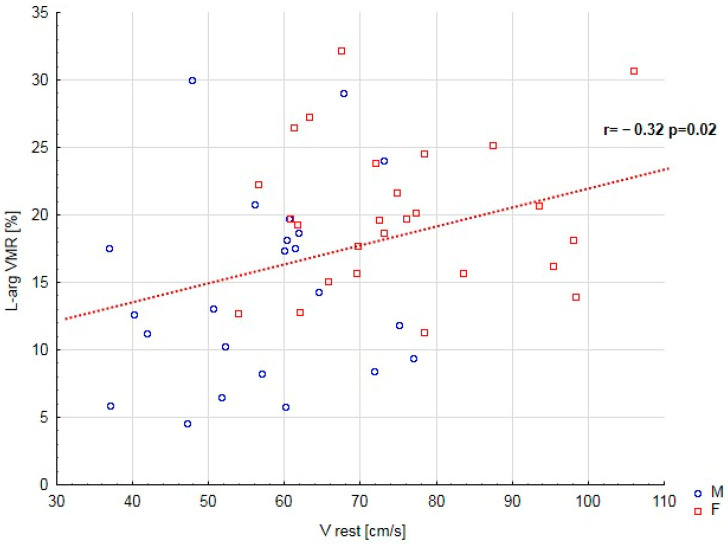
Correlations between V rest and L–arg VMR. V rest—Middle cerebral artery mean flow velocity before L–arginine infusion; L–arg VMR—vasomotor reactivity to L–arginine.

Additionally, a positive correlation was found between HbA_1C_ and BMI and TG serum level, between LDL serum level and cigarette smoking load and TG serum level, and between TG serum level and BMI. The study revealed negative correlations between patients’ age and L–arg VMR, age at onset, and diabetes duration. In addition, the study found a negative correlation between TG serum level and T1D duration and between L–arg VMR and V rest (Figure 1). There was a positive correlation between HbA1C levels and both BMI and triglyceride serum levels. Additionally, a positive relationship was observed between LDL serum levels, cigarette smoking load, and TG serum levels, as well as between TG serum levels and BMI. On the other hand, the study revealed negative correlations: as patients’ age increased, there was a corresponding decrease in L–arginine VMR. Furthermore, the age at onset and the diabetes duration showed negative correlations with patients’ age. Finally, the research identified a negative correlation between TG serum levels and T1D duration (Table 2).

### 2.3. Co-Variated Comparisons Between Females and Males

**Figure 2:**
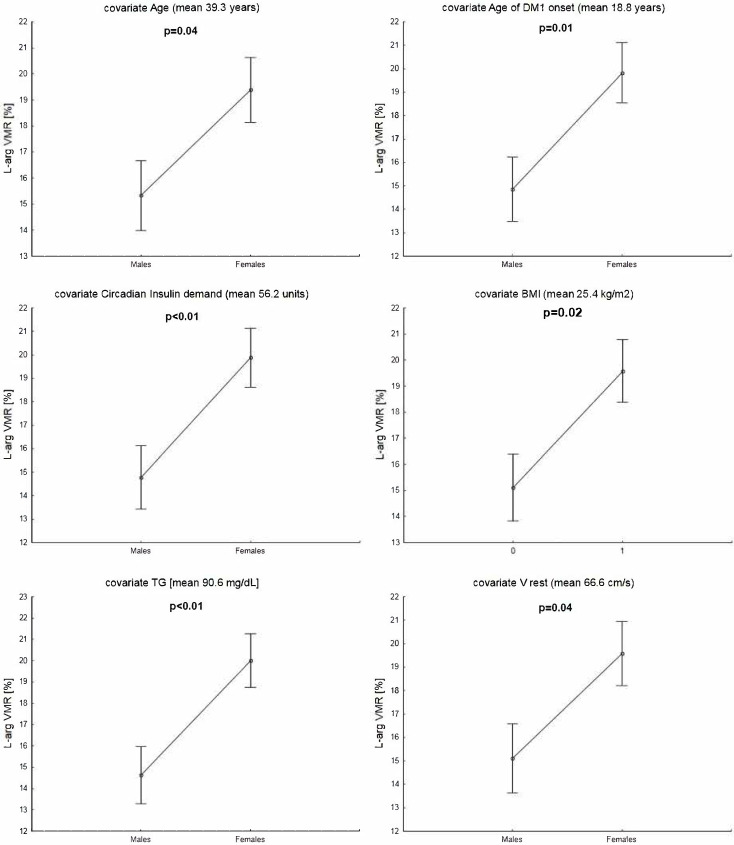
ANCOVA comparisons of cerebral vasomotor reactivity to L–arginine between males and females with type 1 diabetes. Data presented as median and range or mean ± SD: SD—standard deviation; TG—triglycerides; BMI—body mass index; V rest—Middle cerebral artery mean flow velocity prior L–arginine infusion; L–arg VMR—vasomotor reactivity to L–arginine.

Given the many correlations between continuous variables in the study group, ANCOVA comparisons of L–arg VMR between males and females were used instead of regression analysis. The findings definitively support the hypothesis that there was a lower L–arginine VMR in males compared to females, after adjusting for covariates such as patients’ age, age at T1D onset, insulin/24 h, TG serum level, BMI, and mean flow velocity before L–arginine infusion (Figure 2).

## 3. Discussion

Our paper is the first to highlight differences in nitric oxide cerebral-induced vasomotor reactivity between females and males with T1D. We found that middle-aged women with T1D exhibited higher cerebral vasomotor reactivity to L–arginine and higher blood flow velocities in the middle cerebral artery compared to men. In addition, the difference in vasomotor reactivity remained significant even after adjusting for baseline flow velocities. The higher L–arg VMR values in females persisted after adjusting for factors such as age, diabetes duration, BMI, or triglyceride levels.

Previous reports examining gender differences in cerebrovascular reactivity primarily focused on carbon dioxide stimulation and predominantly involved healthy subjects. However, these studies have yielded contradictory results. For instance, Carter et al. found that females had greater reactivity of the middle cerebral artery to CO_2_ compared to males [40]. Similarly, research by Kastrup et al., Karnik et al., and Olah et al. demonstrated an increased vasodilatory response to acetazolamide in pre-menopausal women compared to men [41,42,43]. In contrast, Miller et al. reported that young men had a higher cerebral blood flow response to hypercapnia than young women [44]. Moreover, some studies revealed that cerebral microvasculature status may vary significantly during pregnancy or throughout the menstrual cycle due to fluctuations in estrogen levels [45,46,47]. In contrast, Favre et al. observed that young, healthy females demonstrated better cerebral autoregulation compared to males, and this difference remained unaffected by the phases of the menstrual cycle [48]. Similarly, Korad et al. noted a limited impact of the menstrual cycle on cerebral autoregulation [49]. Furthermore, Baranka et al. reported that acute cerebrovascular responses are influenced by contraceptive use and the menstrual cycle [50]. Johnson et al. also noted that the dynamic cerebral vasomotor response to physiological changes in CO_2_ is positively affected by being female [51]. However, Barnes et al. suggested that cyclooxygenase inhibitors may eliminate age-related differences in cerebral vasodilator responses to hypercapnia [52].

Studies on cerebral vasomotor reactivity in patients with T1D published in international journals have not shown any gender-specific effects. However, most of them did not directly compare CVR between men and women with T1D or have not found gender as a cofactor of CVR [1,2,3,4,53]. The unique study that directly compared CVR between women and men with type 1 diabetes, conducted by Fülesdi et al., showed no significant differences [12]. It should be noted that all these studies have assessed CVR induced by changes in CO_2_ partial pressure. Studies using the L–arginine infusion test to assess vasomotor reserve in patients with type 1 diabetes have not been presented in the world literature so far. In the current paper, we confirm the effect of gender on L–arg VMR mentioned in our preliminary univariate analysis [54]. Our results confirm that the use of a method specifically designed to selectively assess the vasodilatory capacity of the vascular endothelium is sensitive to detection of CVR pathology in T1D.

Cerebrovascular reactivity to L–arginine results in increased blood flow in cerebral vessels due to nitric oxide release [35,36,38,55,56]. This contrasts with CO_2_ stimulation techniques that rely on the chemical regulation due to serum partial CO_2_ variations [57]. Whereas reactivity to L–arginine selectively reflects the mechanoregulatory mechanisms of cerebral blood flow driven by endothelial-mediated vasodilation [35,58,59,60]. Previous publications revealed that CVR to L–arginine is significantly impaired in adults with hypertension, irrespective of clinical and morphological evidence of cerebral small vessel disease [39,61]. Moreover, its augmentation has been shown after normalization of elevated cholesterol values with statin therapy in those patients [37]. Referring to gender, Perko et al. found that cerebrovascular reactivity to L–arginine is higher, both in the anterior and posterior territory, in females than in males under healthy conditions [62]. Reduced CVR to L–arginine in the posterior circulation of migraine patients has also been identified as a potential explanation for the increased incidence of cerebral infarcts within the posterior circulation among migraineurs [63].

Previous reports have shown equivocal data regarding the association between gender and microvascular complications in patients with T1D. Takaike et al. demonstrated that female gender serves as a significant risk factor for the development of severe retinopathy requiring photocoagulation; however, it was not found to be a risk factor for albuminuria in a cohort of young Japanese individuals with T1D [15]. Similarly, Bjerg et al. reported a notable correlation between female gender and an increased risk of retinopathy in Scandinavian patients with T1D [17]. Additionally, Benitez-Aguirre has reported that girls may be at an increased risk of developing retinopathy earlier than boys during puberty [64]. On the other hand, Jansson et al. identified male gender as a notable predictor of retinopathy. It is important to note, however, that Laiginhas et al., Joner et al., and Schreur et al. found no significant differences in the prevalence of diabetic retinopathy and nephropathy between young females and males [13,14,16,18]. Regarding other microvascular beds, previous research by Neubauer-Geryk et al. revealed gender-related differences in skin oxygenation among children with uncomplicated type 1 diabetes mellitus [65]. However, our previous study pointed out no association between cutaneous microvascular dysfunction and the presence and severity of cerebral microangiopathy in T1D [5]. Furthermore, there has been no observed correlation between diabetic neuropathy in young patients with type 1 diabetes and reduced cutaneous vascular reactivity [53].

It is important to know certain limitations of our report. Specifically, the phases of the menstrual cycle were not included in the analysis. As a result, we are unable to directly support the hypothesis regarding the protective influence of estrogens on cerebrovascular reactivity, and our findings do not fully address the underlying mechanisms of CVR protection in premenopausal females with type 1 diabetes. To enhance the validity of our conclusions, a functional evaluation of cerebral microvasculature through brain magnetic resonance imaging (MRI) could provide valuable insights. Previous studies have indicated that healthy females demonstrate Higher Blood Oxygenation Level-Dependent (BOLD) cerebrovascular reactivity and increased basal cerebral blood flow (CBF), as observed through arterial spin labeling and four-dimensional MRI, compared to male participants [66]. Additionally, the identification of morphological markers of cerebral microangiopathy within white matter using MRI could further support our hypothesis. A recent study by Tarkkonen et al. found that white matter hyperintensities (WMHs) and cerebral microbleeds often coexist and progress rapidly in middle-aged individuals with type 1 diabetes [8]. Finally, the relatively small sample size may raise concerns regarding statistical power and residual confounding. Therefore, our results can be considered preliminary, and further studies with larger cohorts are warranted.

In summary, we believe that elucidating the influence of gender on the degree of cerebral microangiopathy may be beneficial for developing early stroke prevention strategies in patients with type 1 diabetes. The negative impact of male gender on cerebral endothelial function in type 1 diabetes suggests that such strategies should be particularly relevant for biological men with type 1 diabetes. Given the results of previous studies with L–arginine, both statin therapy and tight blood pressure control should be included in practical recommendations for primary cerebrovascular prevention in these patients [37,61]. This conclusion can also theoretically be extrapolated to other populations, as numerous studies demonstrate a different cerebrovascular risk between middle-aged Caucasian men and women in the general population [19,20,21,22].

## 4. Methods and Materials

The study group included 49 patients with type 1 diabetes, including 26 women and 23 men. These participants were recruited from the Regional Diabetology Centre of the Medical University of Gdansk (Table 1). Selection criteria included a minimum duration of diabetes of six years and the absence of focal neurologic deficits.

The exclusion criteria were established with a precise definition to ensure the study’s integrity. Participants with significant stenosis or occlusion of extracranial arteries, as determined by carotid Doppler ultrasound, were excluded from participation.

The additional exclusion criteria encompassed a medical history of cerebrovascular events, including stroke and transient ischemic attack, as well as a diagnosis of coronary heart disease, diabetic foot complications, orthostatic hypotension, or significant head trauma. Additionally, individuals with renal insufficiency, a history of rheumatologic diseases, and those who were pregnant, postmenopausal, or utilizing estrogen therapies, whether oral or transdermal, were excluded from participation. All examinations were systematically conducted between 8:00 A.M. and 1:00 P.M. Study participants received detailed instructions to refrain from consuming coffee and tobacco on the examination day and to avoid sleep deprivation in the days prior. The study protocol encompassed a comprehensive medical history, a neurological examination, fundoscopy, laboratory testing, and carotid and transcranial Doppler ultrasonography. The Medical Ethics Committee of the Medical University of Gdansk has approved the study protocols (decisions: NKEBN/335/2008 and NKEBN/335–60/2009). Informed consent was obtained from all participants prior to their involvement in the study, ensuring their comprehension and consent to participate.

### 4.1. Subject Characteristics

Patient histories were obtained, encompassing information regarding previous and current disorders, co-morbid conditions, and tobacco usage. Patients’ weight and height were measured, followed by the calculation of their body mass index. Overweight was classified as a BMI exceeding 25 kg/m^2^, while obesity was defined as a BMI exceeding 30 kg/m^2^. Blood pressure was monitored in all subjects using a conventional sphygmomanometer. A diagnosis of arterial hypertension was established if two consecutive measurements indicated a systolic blood pressure exceeding 140 mm Hg or a diastolic blood pressure exceeding 90 mm Hg, or if the individual was taking antihypertensive medication.

Laboratory tests for patients with type 1 diabetes encompassed evaluations of microalbuminuria, albuminuria/creatinine index, total serum cholesterol, LDL cholesterol, HDL cholesterol, triglycerides, and HbA_1C_. Blood and urine samples were obtained on the day of the examination. The analysis of albumin secretion was conducted using three measurements taken closest to the study date. According to the guidelines established by the American Diabetes Association, hyperlipidemia was diagnosed if total cholesterol was >175 mg/dL and/or triglycerides were >150 mg/dL and/or LDL cholesterol was >100 mg/dL. Additionally, hyperlipidemia may be identified if HDL cholesterol levels are below 40 mg/dL for males or below 50 mg/dL for females, or if an individual is undergoing treatment with medications designed to reduce cholesterol or triglyceride levels [67].

The diagnosis of diabetic nephropathy was made on the basis of the presence of microalbuminuria or overt nephropathy. The European Association for the Study of Diabetes has established criteria for the diagnosis of microalbuminuria. According to these criteria, the presence of microalbuminuria is indicated by an albumin-to-creatinine ratio greater than 30 mg of albumin per mg of creatinine in a random spot urine collection or less than 20 mg of albumin per minute in an overnight urine collection. This diagnosis must be confirmed in two out of three urine collections conducted at intervals of up to six months [68].

Retinopathy was identified through fundoscopy performed by an ophthalmologist certified by the Polish Ophthalmological Society. The severity of the retinopathy was classified based on the stages of diabetic retinopathy as outlined by the American Academy of Ophthalmology. Furthermore, a documented history of prior photocoagulation therapy was identified as a significant predictor of diabetic retinopathy [69].

The diagnosis of diabetic neuropathy was made in accordance with the established criteria delineated by the Neurologic Symptom Score and Neurologic Disability Score. These criteria were met based on a comprehensive evaluation of patient symptoms and the identification of neuropathic deficits during a neurologic examination [70].

### 4.2. Transcranial Doppler Examination

Middle cerebral artery flow parameters were measured by TCD through the temporal bone window, using a MultiDop T2 DWL device (DWL Elektronische Systeme, Singen, Germany) equipped with two 2 MHz probes, a fixation headband, and monitoring software (version MF 8.27 l; DWL Elektronische Systeme, Singen, Germany). Flow measurements were performed simultaneously in both middle cerebral arteries by a single, well-trained ultrasonographer (GK). In five patients, the signal was obtained unilaterally due to a weak acoustic window on the contralateral side. One patient was excluded from the study due to the lack of an acoustic window on both sides. Measurements were performed in the supine position after 10 min of rest. Before the tests, systemic blood pressure was measured. Median values of the arithmetic mean of velocity measurements prior to and 10 min after intravenous infusion of L–arginine (30 g of L-arginine-hydrochloride 21%, Braun, Melsungen, Germany) continuously infused by pump for 30 min from both middle cerebral arteries were obtained for further off-line analyses. Vasomotor reactivity to L–arginine was calculated using the following formula [35]:

L–arg VMR = ((V L–arg − V rest)/V rest) × 100%

(L–arg VMR—vasomotor reactivity to L–arginine; V L–arg—mean middle cerebral arteries flow velocity after 10 min of L–arginine infusion, V rest—mean middle cerebral arteries flow velocity prior to L–arginine infusion).

### 4.3. Statistical Analysis

All analyses were conducted using STATISTICA version 9.1 software (StatSoft Inc., Tulsa, OK, USA). Shapiro–Wilk tests were performed to evaluate the distribution of continuous variables. Differences between groups were analyzed using Student’s *t*-test for normally distributed variables (age at T1D onset, T1D duration, HbA_1_c, insulin/24 h, mean blood pressure, LDL, V rest, L–arg VMR) or with the Mann–Whitney U test for the other variables (age, BMI, TG, cigarette smoking load). The chi2 test was used to compare the prevalence of systemic microvascular complications and concomitant risk factors in various groups. The correlation was assessed by the Spearman rank correlation test. Co-variated comparisons of L–arg VMR were made with the ANCOVA test. The level of *p* < 0.05 was regarded as statistically significant.

## 5. Conclusions

The current findings indicate an association between female gender and increased reactivity to L–arginine in middle-aged individuals with T1D. This observation may suggest that middle-aged males with T1D are more prone to cerebral endothelial impairment. This underscores the importance of implementing cerebrovascular prevention strategies at an early stage in the management of patients with T1D, with a particular focus on male patients.

## Figures and Tables

**Table 1:** Characteristics of patients with type 1 diabetes.

	Males *n* = 23	Females *n* = 26	*p*
Age [years]	39.7 (31.3–55.7)	36.5 (25.0–45.5)	0.02
Age onset [years]	21.9 ± 7.7	16.1 ± 7.3	<0.01
Type 1 diabetes duration [years]	20.5 ± 7.1	21.3 ± 6.6	0.70
HbA_1C_ [%]	7.9 ± 0.9	7.9 ± 1.4	0.87
Insulin/24 h [units]	61.6 ± 17.0	51.1 ± 16.1	0.04
Arterial hypertension [%]	30.4	15.4	0.21
ACEI or ARB treatment [%]	21.7	11.5	0.33
Mean arterial blood pressure [mm Hg]	88.4 ± 12.3	92.0 ± 16.1	0.39
Hyperlipidemia [%]	82.6	76.9	0.62
Statin treatment [%]	26.1	19.2	0.56
TG [mg/dL]	75 (48–299)	66 (39–380)	0.03
LDL [mg/dL]	118.3 ± 28.4	112.0 ± 30.8	0.49
Overweight or obesity [%]	69.6	30.8	0.01
BMI [kg/m^2^]	26.9 (21.5–32.6)	23.4 (19.0–37.3)	<0.01
Cigarette smoking [%]	21.7	26.9	0.67
Cigarette smoking load [pack-years]	0 (0.0–20.0)	0 (0–15)	0.99
Diabetic nephropathy [%]	39.1	30.8	0.53
Diabetic retinopathy [%]	52.2	46.1	0.67
Diabetic neuropathy [%]	60.9	46.1	0.32
V rest [cm/s]	60.0 (36.8–77.0)	72.7 (53.8–106.0)	<0.01
L–arg VMR [%]	14.6 ± 7.1	20.1 ± 5.4	<0.01

Data presented as median and range or mean ± SD: SD—standard deviation; HbA_1C_—glycated hemoglobin; h—hours; ACEI—angiotensin-converting enzyme inhibitor; ARB—angiotensin receptor blocker; TG—triglycerides; LDL—low density lipoprotein; BMI—body mass index; L–arg VMR—vasomotor reactivity to L–arginine.

**Table 2:** Correlations between clinical and laboratory parameters and L–arg VMR.

	Age	Age of Onset	T1D Duration	Insulin/24 h	HbA_1C_	LDL	TG	BMI	Cigarette Smoking Load	V Rest
Age of onset	0.48	-	-	-	-	-	-	-	-	-
	<0.01	-	-	-	-	-	-	-	-	-
T1D duration	0.14	−0.68	-	-	-	-	-	-	-	-
	0.32	<0.01	-	-	-	-	-	-	-	-
insulin/24 h	0.02	0.23	−0.25	-	-	-	-	-	-	-
	0.91	0.12	0.08	-	-	-	-	-	-	-
HbA_1C_	−0.08	−0.01	−0.10	0.42	-	-	-	-	-	-
	0.57	0.94	0.48	<0.01	-	-	-	-	-	-
LDL	−0.21	−0.19	0.12	0.23	0.10	-	-	-	-	-
	0.15	0.19	0.43	0.12	0.49	-	-	-	-	-
TG	0.05	0.32	−0.28	0.51	0.41	0.43	-	-	-	-
	0.72	0.02	0.05	<0.01	<0.01	<0.01	-	-	-	-
BMI	0.31	0,2	<−0.01	0.55	0.40	−0.02	0.34	-	-	-
	0.03	0.16	0.99	<0.01	<0.01	0.91	0.02	-	-	-
Cigarette smoking load	0.54	0.17	−0.13	0.17	0.15	0.31	0.30	−0.06	-	-
	0.71	0.23	0.35	0.25	0.32	0.03	0.03	0.67	-	-
V rest	−0.24	−0.11	−0.10	−0.11	<−0.11	<0.01	<0.12	−0.15	0.10	-
	0.09	0.43	0.47	0.43	0.98	0.99	0.40	0.31	0.51	-
L–arg VMR	−0.34	−0.23	−0.13	−0.18	−0.19	0.23	−0.14	−0.43	0.09	0.32
	0.01	0.11	0.38	0.22	0.20	0.11	0.32	<0.01	0.52	0.02

Data presented as median and range or mean ± SD: SD—standard deviation; HbA_1C_ T1D—type 1 diabetes; HbA_1C_—glycated hemoglobin; TG—triglycerides; LDL—low density lipoprotein; BMI—body mass index; V rest—Middle cerebral artery mean flow velocity before L–arginine infusion; L–arg VMR—vasomotor reactivity to L–arginine.

## Data Availability

The datasets generated during and/or analyzed in the current study are available from the corresponding author upon reasonable request.
